# Occupational role and Covid-19 among foreign-born healthcare workers: a registry-based study

**DOI:** 10.1093/eurpub/ckac130.175

**Published:** 2022-10-25

**Authors:** C Nwaru, H Li, C Bonander, A Santosa, S Franzén, M Rosvall, F Nyberg

**Affiliations:** 1 School of Public Health and Community Medicine, Institute of Medicine, Sahlgrenska Academy, University of Gothenburg, Gothenburg, Sweden; 2 Primary Heath Care, Regionhälsan, Region Västra Götaland, Gothenburg, Sweden

## Abstract

**Background:**

Studies have shown an elevated risk of Covid-19 among foreign-born healthcare workers (HCWs), but data on the distribution of the risk in different occupational roles are lacking. Such data are needed for the effective control of Covid-19 risk among HCWs. Here, we examined the risk of Covid-19 infection and hospitalization in foreign-born HCWs in different occupational roles in Sweden.

**Methods:**

We prospectively linked occupational data (2018-2019) of 783950 employed foreign-born (20-65 years) workers to Covid-19 data registered between 1 January 2020 and 30 September 2021. We used Cox proportional hazards regression to estimate the risk of Covid-19 infection and hospitalization in foreign-born HCWs in eight different occupational groups compared to non-HCWs, and to assess whether the associations varied by region of birth. The analyses were adjusted for sociodemographic and socioeconomic factors, comorbidities, and Covid-19 vaccination.

**Results:**

All HCWs had a higher risk of Covid-19 outcomes than non-HCWs, but the risk differed by occupational role. Assistant nurses had the highest risk both for Covid-19 infection (HR 1.80; 95%CI 1.74-1.87) and hospitalization (HR 1.85; 95%CI 1.57-2.18); other allied HCWs had the lowest risk (infection: HR 1.23; 95%CI 1.11-1.36; hospitalization: HR 1.02; 95%CI 0.63-1.67)). In some healthcare occupations, the relative risk of Covid-19 varied by region of birth. For example, physicians and dental nurses/hygienists of African and Asian origin had a higher risk of Covid-19 infection than European-born in the same occupation. In contrast, European-born assistant nurses had a greater risk of both outcomes than non-European-born in the same occupation.

**Conclusions:**

The risk of Covid-19 among foreign-born HCWs varied by occupational role and region of birth. Public health efforts that target occupational exposures as well as incorporate culturally responsive measures may help to reduce Covid-19 risk among foreign-born HCWs.

**Key messages:**

- Risk of Covid-19 among foreign-born healthcare workers varied by occupational role and region of birth.
- Multiple approaches are needed to protect this population group against Covid-19.

